# Influence of variability in the cyclooxygenase pathway on cardiovascular outcomes of nephrosclerosis patients

**DOI:** 10.1038/s41598-022-27343-z

**Published:** 2023-01-23

**Authors:** Luz M. González, Nicolás R. Robles, Sonia Mota-Zamorano, José M. Valdivielso, Laura González-Rodríguez, Juan López-Gómez, Guillermo Gervasini

**Affiliations:** 1 Department of Medical and Surgical Therapeutics, Medical School, University of Extremadura, Av. Elvas S/N 06071, Badajoz, Spain; 2 Service of Nephrology, Badajoz University Hospital, Badajoz, Spain; 3 ISCIII RICORS2040, Madrid, Spain; 4 Vascular and Renal Translational Research Group, UDETMA, IRBLleida, Lleida, Spain; 5 Service of Clinical Analyses, Badajoz University Hospital, Badajoz, Spain; 6 Institute of Molecular Pathology Biomarkers, University of Extremadura, Badajoz, Spain

**Keywords:** Medical genetics, Nephrology, Kidney diseases, Medical research, Genetics research, Risk factors, Cardiovascular diseases, Chronic kidney disease

## Abstract

Nephrosclerosis patients are at an exceptionally high cardiovascular (CV) risk. We aimed to determine whether genetic variability represented by 38 tag-SNPs in genes of the cyclooxygenase pathway (*PTGS1, PTGS2*, *PTGES*, *PTGES2* and *PTGES3*) leading to prostaglandin E2 (PGE2) synthesis, modified CV traits and events in 493 nephrosclerosis patients. Additionally, we genotyped 716 controls to identify nephrosclerosis risk associations. The addition of three variants, namely *PTGS2* rs4648268, *PTGES3* rs2958155 and *PTGES3* rs11300958, to a predictive model for CV events containing classic risk factors in nephrosclerosis patients, significantly enhanced its statistical power (AUC value increased from 78.6 to 87.4%, p = 0.0003). Such increase remained significant after correcting for multiple testing. In addition, two tag-SNPs (rs11790782 and rs2241270) in *PTGES* were linked to higher systolic and diastolic pressure [carriers vs. non-carriers = 5.23 (1.87–9.93), p = 0.03 and 5.9 (1.87–9.93), p = 0.004]. *PTGS1*(COX1) rs10306194 was associated with higher common carotid intima media thickness (ccIMT) progression [OR 1.90 (1.07–3.36), p = 0.029], presence of carotid plaque [OR 1.79 (1.06–3.01), p = 0.026] and atherosclerosis severity (p = 0.041). These associations, however, did not survive Bonferroni correction of the data. Our findings highlight the importance of the route leading to PGE2 synthesis in the CV risk experienced by nephrosclerosis patients and add to the growing body of evidence pointing out the PGE2 synthesis/activity axis as a promising therapeutic target in this field.

## Introduction

Nephrosclerosis is an umbrella term that usually denotes the presence of renal impairment in an aging patient with hypertension, frequently with no histological confirmation^1^. This is a chronic kidney disease (CKD) that not only contributes greatly to progression to end-stage kidney disease (ESKD), but that also have an immense impact on global cardiovascular (CV) risk^2^. Classic CKD biomarkers only stand out when the disease is well under way and there is therefore a need for novel markers that can help in the early identification of patients at risk for adverse outcomes.

Arachidonic acid is metabolized by cyclooxygenases COX1 and COX2 to a variety of inflammatory mediators. Of these, PGE2 is the major arachidonic metabolite in the kidney^3^ and is responsible for both renal homeostasis and pathological mechanisms such as inflammation, hyperfiltration, fibrosis, apoptosis or renin-angiotensin aldosterone system (RAAS) activation^4–6^. PGE2 production is directly governed by one cytosolic (cPGES) and two microsomal synthases (mPGES1 and mPGES2), whose activity has been related to renal function impairment and blood pressure elevation^7,8^.

**Figure 1:**
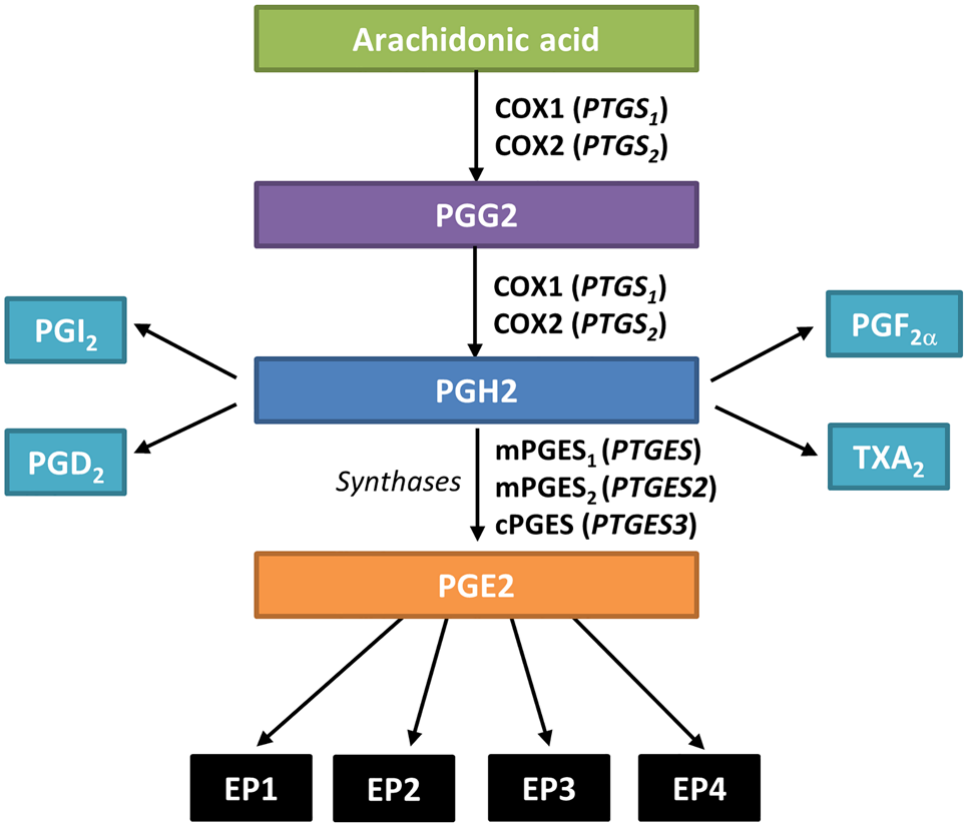
Overview of the cyclooxygenase-PGE2 pathway. Gene names are shown in parenthesis.

Genetics are known to play a role in CKD onset and development^9,10^, accordingly, we hypothesize that the pathway leading to PGE2 synthesis and its actions may be a suitable candidate for identifying genetic variants relevant for CKD, particularly for its CV-associated impact. Indeed, we have recently shown how single nucleotide polymorphisms (SNPs) in the genes coding for PGE2 receptors^11^ and phospholipase-related genes^12^ are associated with CV outcomes in these patients. Our aim was therefore to identify tag-SNPs (variants that represent variability in a certain region of the gene locus) in five candidate genes of the cyclooxygenase pathway (Fig. 1), namely *PTGS1* (COX1), *PTGS2* (COX2), *PTGES* (mPGES1), *PTGES2* (mPGES2) and *PTGES3* (cPGES), and investigate putative associations with CV traits and events (CVE) in these patients. Additionally, we screened a group of individuals with normal renal function to identify associations with the risk of nephrosclerosis.

## Results

Median age and interquartile range (IQR) of patients and controls were, respectively, 66 (12) and 60 (17) years, whilst the percentage of males in the same groups were 50.2% and 67.7%. The percentage of classic CV risk factors such as diabetes, hypertension, or dyslipidemia, was higher in the CKD patients than in the control group (p < 0.0001). These variables were therefore included, amongst others, in the regression models that were later applied. In contrast, the frequency of smokers was similar in both study groups. As expected, biochemistry data were also significantly different between patients and controls. Table 1 shows these and other demographic and clinical characteristics of the study groups stratifying patients by CKD stage.

**Table 1:** Demographic and clinical parameters of the study population.

	Control (N = 716)	CDK3 (N = 307)	CDK4-5 (N = 123)	CDK 5D (N = 63)	p-value (control vs. CKD)	p-value (CKD groups)
Males (%)	359 (50.2)	211 (68.7)	80 (65)	43 (68.3)	< 0.0001	NS
Age (years)	60 (17)	67 (10)	64 (14)	61 (18)	< 0.0001	< 0.001
**Ethnicity**						
Caucasian	709 (99)	304 (99)	120 (97.6)	58 (92.1)	< 0.01	< 0.01
Other	7 (1)	3 (1)	3 (2.4)	5 (7.9)		
Weight (kg)	75.15 (19.83)	80.25 (16.55)	78.60 (19.70)	75 (21.2)	< 0.0001	< 0.05
BMI	28.25 (5.13)	29.97 (5.77)	28.75 (7.32)	26.96 (6.83)	< 0.0001	< 0.01
Glucose (mg/dL)	97 (20)	102.5 (25)	101.00 (21)	89 (21)	< 0.001	< 0.0001
Total cholesterol (mg/dL)	200.7 (43)	185 (54)	175.50 (55)	160.5 (48)	< 0.0001	< 0.0001
Cholesterol HDL (mg/dL)	52 (19.2)	47 (17)	46.9 (18.2)	45 (17)	< 0.0001	NS
Cholesterol LDL (mg/dL)	126.4 ± 32.24	110.81 ± 34.47	100.79 ± 34.73	89.55 ± 34.67	NS	NS
Calcium (mg/dL)	9.4 (0.5)	9.50 (0.6)	9.35 (0.8)	9.15 (0.8)	NS	< 0.0001
Potassium (mEq/L)	4.5 (0.5)	4.65 (0.6)	4.8 (0.8)	4.86 (0.6)	< 0.0001	< 0.05
Sodium (mEq/L)	141 (3)	141 (3)	141 (4)	138.5 (4)	NS	< 0.0001
ACR (mg/g)	6.4 (37.2)	37.11 (187.62)	212.11 (554.99)	–	< 0.0001	< 0.0001
eGFR (ml/min/1.73 m^2^)	88.82 (21.34)	44 (13.74)	20.67 (9)	–	< 0.0001	< 0.0001
**Cardiovascular events**						
Yes	9 (1.3)	25 (8.1)	10 (8.1)	6 (9.5)	< 0.0001	NS
No	707 (98.7)	282 (91.9)	113 (91.9)	57 (90.5)		
**Hypertension**						
Yes	405 (56.6)	293 (95.4)	121 (98.4)	60 (95.2)	< 0.0001	NS
No	310 (43.4)	14 (4.6)	2 (1.6)	3 (4.8)		
**DM**						
Yes	55 (7.7)	74 (42.1)	32 (26)	8 (12.7)	< 0.0001	NS
No	660 (92.3)	233 (75.9)	91 (74)	55 (87.3)		
**Smoking**						
Non-smoker	327 (45.7)	124 (40.4)	53 (43.1)	26 (41)	NS	NS
Former-smoker	263 (36.8)	130 (42.3)	48 (39)	24 (38.1)		
Current-smoker	125 (17.5)	53 (17.3)	22 (17.9)	13 (20.6)		
Systolic pressure (mmHg)	133 (24)	145 (28)	146 (28)	139 (25)	< 0.0001	NS
Diastolic pressure (mmHg)	80 (14)	81 (14)	79 (15)	80 (20)	NS	NS
Pulse pressure (mmHg)	52 (16)	64 (26)	64 (22)	60 (20)	< 0.0001	NS

This same population has also been analyzed in previous studies by our group^11,12^.

*BMI* body mass index, *eGFR* estimated glomerular filtration rate, *DM* diabetes mellitus, *ACR* albumin-to-creatinine ratio, *NS* not significant.

*PTGES3* rs78343990 showed a significant deviation from the Hardy–Weinberg equilibrium (p < 0.05) and its results were therefore disregarded. Minor allele frequencies (MAF) and successful genotyping rates for the analyzed loci ranged from 2.8 to 38.8% and from 95.2 to 99.8%, respectively.

### Genetic associations with cardiovascular events

A four-year follow-up [median (IQR) = 47 (6) months] was carried out in the population of study that registered 41 and 9 CVE in the CKD patients (8.3%) and control group (1.3%), respectively. As expected, nephrosclerosis had a deep impact on CV risk [OR 7.13 (3.4–14.8), p < 0.0001]. Main features of individuals with and without CVE are listed in Table 2.

**Table 2:** Characteristics of individuals with and without cardiovascular events registered during the follow-up in the whole population of study.

	No CVE	CVE	p-value
Males (%)	300 (66.4)	34 (82.9)	< 0.05
Age (years)	66 (12)	67 (9)	< 0.05
**Ethnicity**			
Caucasian	441 (97.6)	41 (100)	NS
Other	11 (2.4)	–	
Weight (kg)	79.8 (18.40)	81.5 (20.6)	NS
BMI	29.39 (6.07)	29.74 (6.99)	NS
Glucose (mg/dL)	100.5 (24)	102 (27)	NS
Total cholesterol (mg/dL)	181 (54)	165.5 (73)	NS
Cholesterol HDL (mg/dL)	47 (17)	41.55 (21.3)	< 0.01
Cholesterol LDL (mg/dL)	103 (45)	96.5 (56.5)	NS
Calcium (mg/dL)	9.4 (0.6)	9.5 (0.6)	NS
Potassium (mEq/L)	4.7 (0.7)	4.8 (0.6)	NS
Sodium (mEq/L)	141 (3)	141 (3)	NS
Albumin/creatinine (mg/g)	68.87 (284.37)	173.62 (445.07)	NS
eGFR (ml/min/1.73 m^2^)	38.21 (21.02)	37.43 (22)	NS
**Hypertension**			
Yes	435 (96.2)	39 (95.1)	NS
No	17 (3.8)	2 (4.9)	
**DM**			
Yes	100 (22.1)	14 (34.1)	NS
No	352 (77.9)	27 (65.9)	
**Smoking**			
Non-smoker	190 (42)	13 (31.7)	NS
Former-smoker	185 (40.9)	17 (41.5)	
Current-smoker	77 (17)	11 (26.8)	
Systolic pressure (mmHg)	144 (28)	145 (28)	NS
Diastolic pressure (mmHg)	81 (15)	77.50 (18)	NS
Pulse pressure (mmHg)	62 (24)	67 (21)	NS

*BMI* body mass index, *eGFR* estimated glomerular filtration rate, *DM* diabetes mellitus, *NS* not significant.

**Figure 2:**
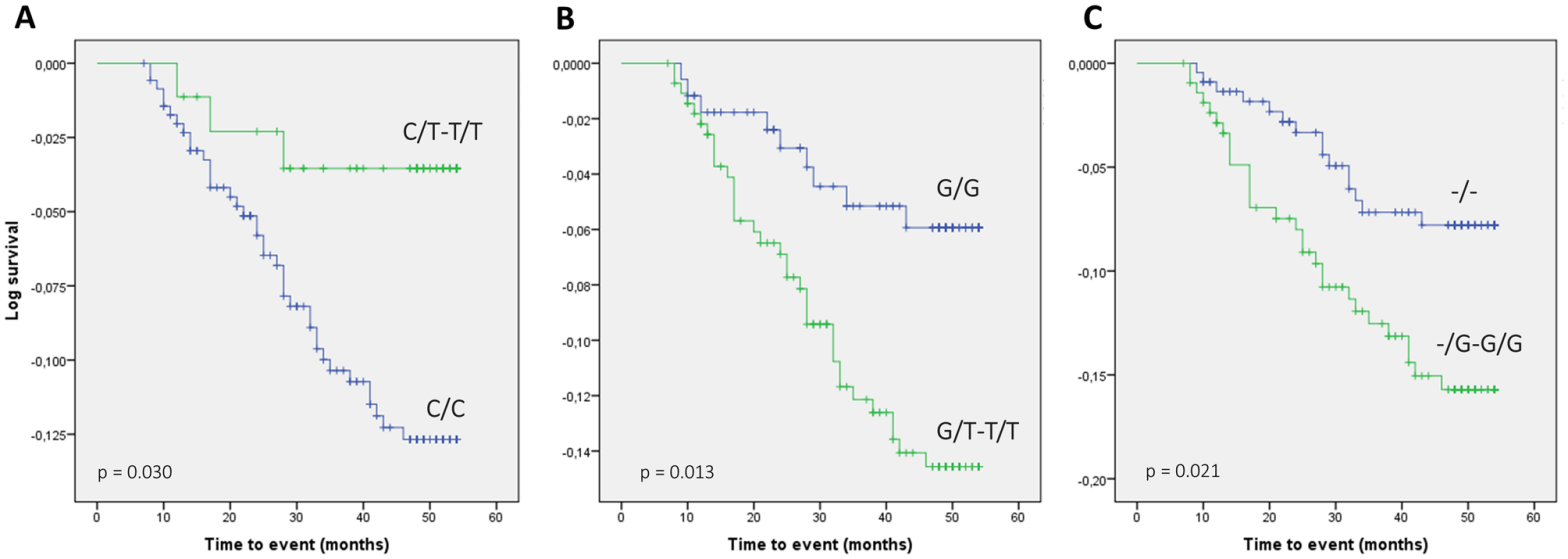
Kaplan–Meier curves for the association of *PTGS2* rs4648268 (**A**), *PTGES3* rs2958155 (**B**) and *PTGES3* rs11300958 (**C**) with cardiovascular event-free survival. Wild type homozygous vs. variant genotypes are depicted. P-values for the long-rank tests carried out for comparison of the different genotypes are shown. The rs11300958 polymorphism produces an insertion (G-allele).

We analyzed the effect of the 38 tag-SNPs studied on CV event-free survival in the patients’ cohort. Kaplan–Meier curves compared with the log-rank test showed that three SNPs, namely *PTGS2* rs4648268, *PTGES3* rs2958155 and *PTGES3* rs11300958, displayed suggestive associations (Fig. 2). Median survival for carriers vs. non-carriers of the three variants were, respectively, 52.79 (51.42–54.16) vs. 50.56 (49.47–51.64) months, p = 0.030; 49.97 (48.65–51.28) vs. 52.59 (51.15–53.42) months, p = 0.013; and 49.58 (48.00–51.17) vs. 51.86 (50.78–52.94) months, p = 0.021. The direction of the associations was maintained when analyses were adjusted by traditional CV risk factors (age, sex, BMI, diabetes, hypertension, and CKD stage) in Cox regression models, although corrected p-values were higher than the Bonferroni threshold. Hazard ratios for the variant genotypes were 0.31 (0.09–0.99), p = 0.049; 2.41 (1.15–5.04), p = 0.02 and 2.20 (1.16–4.18), p = 0.016 for rs4648268, rs2958155 and rs11300958, respectively. Supplementary Tables S1–S3 show the resulting Cox models for each SNP. We also performed a sub-analysis to re-assess these associations when only coronary events were considered. Survival analysis show that only PTGS2 rs4648268 remained linked to this subgroup of events (p = 0.020), as none of the patients carrying the T-variant allele experienced an event (Supplementary Fig. S1).

**Figure 3:**
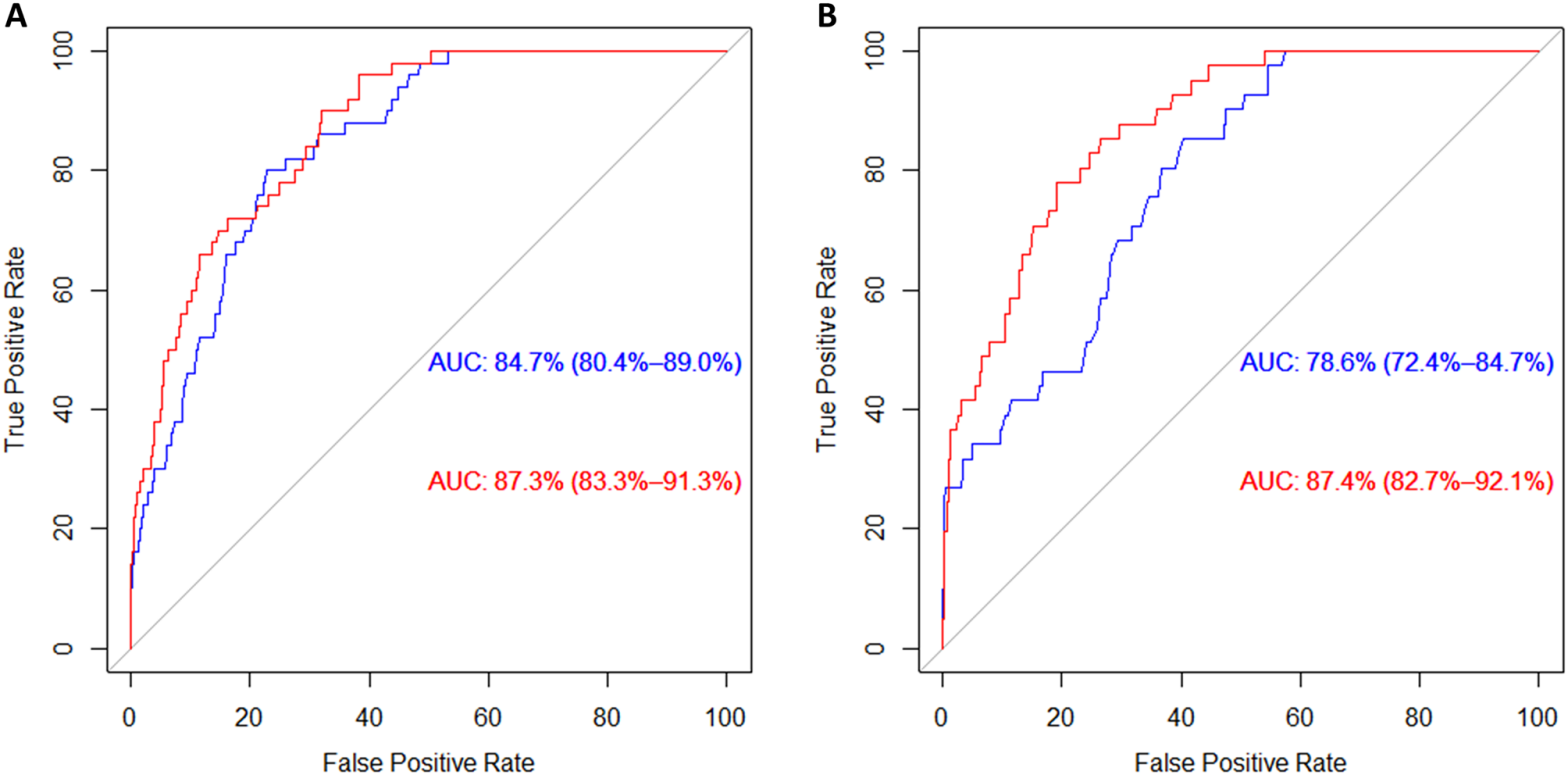
Receiving operating curves for the risk of cardiovascular events in (**A**) the whole population of patients and controls and (**B**) nephrosclerosis patients. The blue line corresponds to the model with classic risk factors and the red line corresponds to the same model when genetic information is added. *AUC* area under the curve.

Next, we developed CV risk prediction models using Receiving Operating Characteristic curves (ROC) analysis, with the state variable being the occurrence of a CV event during follow-up. Figure 3A shows that, in the whole population of study, the addition of the aforementioned three SNPs to a model containing traditional CV risk factors (age, sex, hypertension, diabetes, ethnicity and CKD stage), slightly improved its predictive power from an AUC of 84.7% to 87.3% (p = 0.031). Interestingly, when the analysis was restricted to the patients’ group, this improvement was far larger, as the addition of genetic information made the AUC increase from 78.6 to 87.4% (p = 0.0003, Fig. 3B). Such increase was still significant after Bonferroni correction for multiple testing.

**Figure 4:**
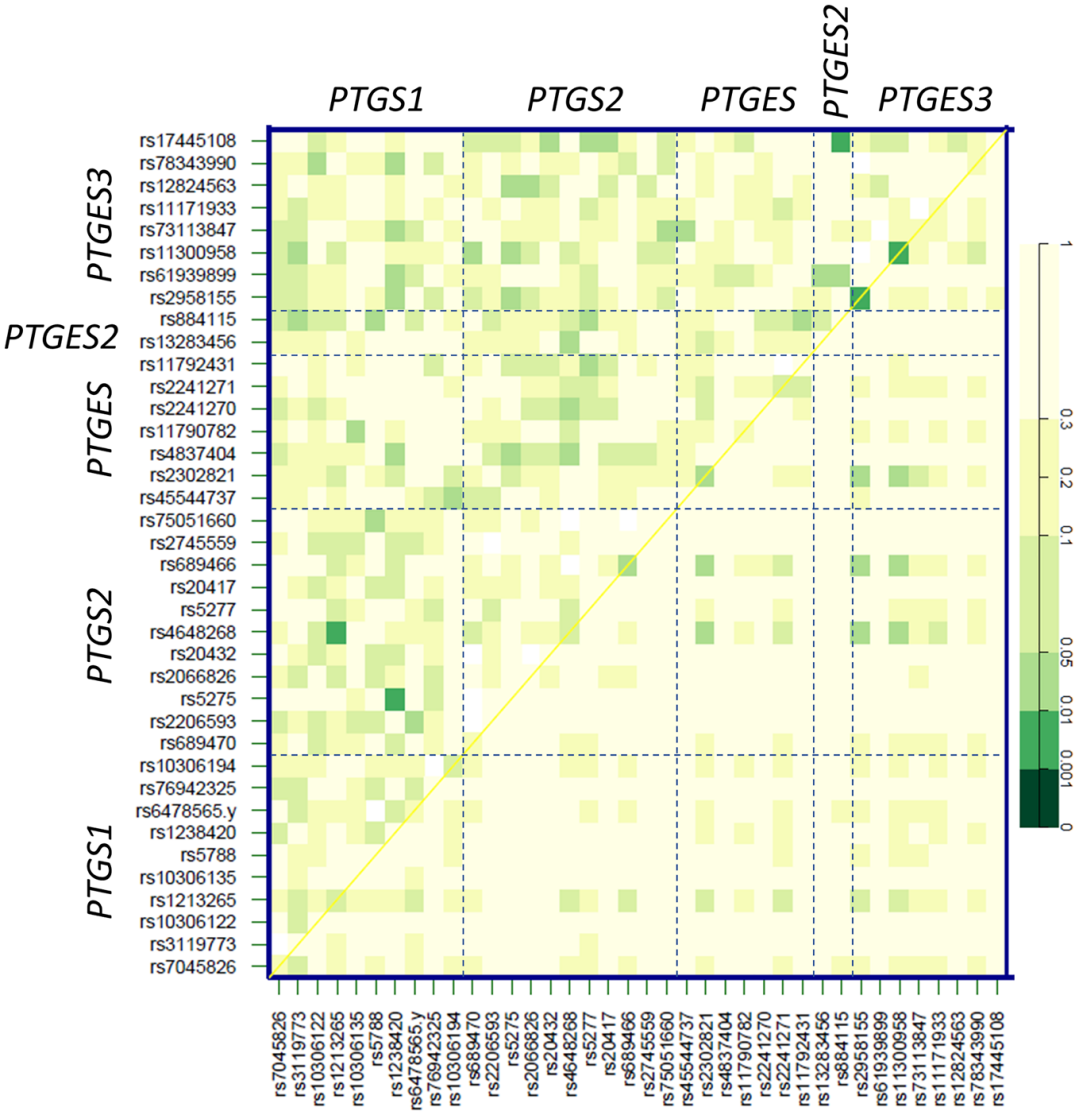
Interactions between genetic variants in the cyclooxygenase-PGE2 pathway and their association with cardiovascular events in nephrosclerosis patients. The upper triangle in the matrix contains p-values for the interaction (epistasis) log-likelihood ratio test. The lower triangle contains p-values from likelihood ratio test comparing the two-SNP additive likelihood to the best of the single-SNP models.

In order to investigate whether any of the 38 tag-SNPs could interact with each other to modify CV risk, we also estimated associations of pairwise statistical epistasis (Fig. 4). Three SNP-pair interactions were identified, two between variants in *PTGS2* (COX2) and *PTGS1* (COX1), namely rs4648268-rs1213265 (p < 0.01) and rs5275-rs1238420 (p < 0.01), and one between SNPs located in PGE2 synthases: *PTGES2* rs17445108-*PTGES3* rs884115 (p < 0.01).

### Genetic associations with cardiovascular-related variables of nephrosclerosis patients

The results of the association analyses between the 38 studied SNPs and blood pressure (BP) revealed that, most notably, two consecutive SNPs in the *PTGES* gene, which codes for a microsomal PGE2 synthase, rs11790782 and rs2241270, were associated with higher systolic BP (SBP), as revealed by linear regression analyses also considering age, sex, BMI, diabetes, ethnicity, and CKD stage. Mean difference values with 95% confidence interval for carriers vs. non-carriers of the two SNPs were 5.23 (1.87–9.93) mmHg, p = 0.03 and 5.9 (1.87–9.93) mmHg, p = 0.004 (Table 3). Interestingly, these same variant genotypes also increased diastolic BP (DBP) figures [2.86 (0.36–5.37), p = 0.026 and 2.77 (0.64–4.92), p = 0.011; Table 3). Two more SNPs, rs20417 and rs2302821 were also related to SBP and DBP, respectively (Table 3). No associations were observed regarding pulse pressure (data not shown).

**Table 3:** Adjusted genetic association analysis with blood pressure in patients with nephrosclerosis.

	Polymorphism		n (mean, mmHg)	Mean difference	p-value
**SBP**					
*PTGS2*	rs20417	C/C	332 (145.0)	4.28 (0.36 to 8.18)	0.032
		C/G-G/G	150 (148.8)		
*PTGES*	rs11790782	G/G	379 (145.4)	5.23 (0.51 to 9.94)	0.030
		G/A-A/A	90 (150.4)		
*PTGES*	rs2241270	C/C	344 (144.8)	5.9 (1.87 to 9.93)	0.004
		C/T-T/T	139 (149.8)		
**DBP**					
*PTGES*	rs2302821	A/A	392 (81.89)	− 2.89 (− 5.41 to − 0.38)	0.024
		A/C–C/C	88 (79.01)		
*PTGES*	rs11790782	G/G	379 (80.95)	2.86 (0.36 to 5.37)	0.026
		G/A-A/A	90 (82.99)		
*PTGES*	rs2241270	C/C	344 (80.77)	2.77 (0.64 to 4.92)	0.011
		C/T-T/T	139 (82.60)		

*SBP* systolic blood pressure, *DBP* diastolic blood pressure.

Overall, atherosclerosis was far more prevalent in the nephrosclerosis patients than in control subjects. Indeed, patients had significantly higher values of: (i) mean atherosclerosis severity score (1.77 ± 0.71 vs. 1.36 ± 0.82, p < 0.0001); (ii) median (IQR) common carotid intima media thickness (ccIMT) [0.77 (0.22) vs. 0.71 (0.20) mm, p < 0.0001]; (iii) frequency of atheromatous plaques (79.4% vs. 55.9%; p < 0.0001); and (iv) median number of plaques detected [3 (4) vs. 1 (3); p < 0.0001]. The threshold for accelerated ccIMT progression in the nephrosclerosis patients was 0.412 mm/year.

The analysis of genetic associations with atherosclerosis measurements in the CKD group was carried out by linear or binary regression analyses (depending on the measured trait) controlling for age, sex, BMI, hypertension, diabetes, ethnicity, and CKD stage. Most notably, *PTGS1* rs10306194 carriers displayed higher values of three parameters, namely ccIMT progression [OR for accelerated progression = 1.90 (1.07–3.36), p = 0.029], presence of carotid plaque [OR = 1.79 (1.06–3.01), p = 0.026] and atherosclerosis severity score (p = 0.041; Table 4). Four more variants were found to be related to atherosclerosis (Table 4), albeit none of them affected more than one measurement. It should be noted, however, that none of the associations with BP traits or atherosclerosis remained significant after correction for the 37 SNPs analyzed.

**Table 4:** Adjusted genetic association analysis with atherosclerosis measurements in patients with nephrosclerosis.

Progression of ccIMT			SP, n (%)	AP, n (%)	OR	p
*PTGS1*	rs10306194	C/C	155 (73.5)	41 (58.6)	1.90 (1.07–3.36)	0.029
		C/A-A/A	56 (26.5)	29 (41.4)		
*PTGS2*	rs2745559	C/C	133 (63)	54 (77.1)	0.46 (0.24–0.86)	0.012
		C/A-A/A	78 (37)	16 (22.9)		

Carotid plaque			No, n (%)	Yes, n (%)	OR	p
*PTGS1*	rs10306194	C/C	101 (76.5)	182 (64.3)	1.79 (1.06–3.01)	0.026
		C/A-A/A	31 (23.5)	101 (35.7)		
*PTGES3*	rs11300958	–/–	76 (59.8)	129 (47.1)	1.86 (1.15–3.01)	0.009
		–/G-G/G	51 (40.2)	145 (52.9)		

Severity score			N, mean	Mean difference	p	
*PTGS1*	rs10306194	C/C	236 (1.70)	0.15 (0.006–0.29)	0.041	
		C/A-A/A	115 (1.91)			
*PTGES*	rs45544737	G/G	318 (1.75)	0.24 (0.01–0.47)	0.041	
		G/A-A/A	34 (1.97)			
*PTGES3*	rs61939899	T/T	291 (1.80)	− 0.23 (− 0.42 to − 0.038)	0.019	

*SP* slow progression, *AP* accelerated progression.

These parameters have previously been analyzed in the same patients regarding their association with other gene sets in previous studies by our group^11,12^.

### Genetic associations with susceptibility to nephrosclerosis

Finally, we genotyped a control group to perform risk analyses adjusted by significant covariates (see “Methods”) under a dominant model of inheritance, i.e., carriers vs. non-carriers. Three SNPs in *PTGS2* (coding for COX2) were associated with nephrosclerosis. These were rs2066826 [OR 0.72 (0.54–0.98), p = 0.032], rs4648268 [OR 1.46 (1.03–2.07), p = 0.032] and rs20417 [OR 0.70 (0.53–0.93), p = 0.013]. However, the addition of these SNPs to a ROC model containing other CKD risk factors did not improve the AUC of the curve significantly [84.6% (82.4–86.7) vs. 84.9% (82.8–87.0) for the combined standard-genetics model, p = 0.156; Supplementary Fig. S2].

## Discussion

Patients with nephrosclerosis face renal impairment that worsens CV risk, while this increased risk speeds up disease progression. This vicious circle results in these patients not only having far higher CV mortality rates than the general population^13^, but also higher rates than those shown by other CKD groups after dialysis or transplant^14^. This situation makes necessary the identification of new biomarkers that can help with the early identification of index patients.

One of the most interesting findings of the study is that three SNPs, one in COX2 and two in a PGE2 synthase, were independent CVE risk factors in the population of nephrosclerosis patients, as shown by Kaplan–Meier curves and Cox regression models. Furthermore, the addition of these variants to a model containing classic risk factors, significantly improved its predictive power. It should be noted that this improvement was greater in the patient group than when the whole population of controls and patients were considered. Two reasons may explain this. First, the incidence of CVE in controls was very low (only nine cases) and, second, standard CV risk factors are already highly prevalent in all nephrosclerosis patients and, therefore, genetic information can really make a difference in terms of the predictive ability of the model. Indeed, the AUC of the combined standard-genetic model was almost nine points higher. In this regard, we have recently reported similar findings for variants in phospholipase genes^12^, located upstream in the same metabolic pathway leading to PGE2, as well as for SNPs present in PGE2 receptors^11^. When interpreting these findings, it should be taken into consideration that even though rs4648268 in PTGS2, and rs2958155 and rs11300958 in PTGES3 significantly correlated with CV outcomes, causality is still to be confirmed until an explanatory mechanism has been identified. Furthermore, different mechanisms could be involved for each SNP, as it seems to be inferred by the fact that rs4648268 correlated with coronary outcomes, whilst the other two variants did not.

We also performed epistasis analyses that revealed several SNP-pair interactions associated with CV risk in the nephrosclerosis patients. Interestingly, the aforementioned effect of rs4648268 in COX2 on CVE experienced by nephrosclerosis patients was enhanced when the variant occurred in combination with another SNP in COX1. It is somewhat logical that variants affecting genes participating in a certain pathway relevant for a given outcome may interact to enhance the effect produced by a single SNP. Indeed, it has repeatedly been shown how interactions between SNPs in aging pathways and other routes may influence CV risk more profoundly than individual SNPs in those same genes do^15–17^.

With regard to CV traits, two consecutive SNPs in *PTGES*, rs11790782 and rs2241270, were associated with higher SBP and DBP values. These SNPs represent the variability occurring in a central area of the gene locus spanning 8.2 kb. *PTGES* codes for the microsomal prostaglandin E synthase-1, the downstream enzyme responsible for PGE2 synthesis. In this respect, it has been shown that a dysfunction of this gene, as seen in knockout mice, favors the production of PGI2 suggesting rediversion of the accumulated PGH2 substrate (see Fig. 1). In turn, this prevents numerous PGE2-dependent damaging mechanisms, such as vascular remodeling, stiffness, and endothelial dysfunction^18^. Therefore, it is tempting to speculate that variability in the area of *PTGES* tagged by these two variants might result in increased activity/expression of the encoded synthase, thus elevating PGE2 levels and, consequently, leading to higher BP values.

The analysis of associations with atherosclerosis measurements revealed that *PTGS1* rs10306194, tagging the distal region of the gene coding for COX1, was the most relevant SNP, as the variant genotypes were linked to increased presence of plaque in the carotid, higher atherosclerosis severity score and accelerated ccIMT progression. This tag-SNP has not been widely studied, but one report has regarded it as clinically relevant, after being solidly associated with acute urticaria/angioedema^19^. This polymorphism produces an A-to-T change in the 3′-untranslated (UTR) region of *PTGS1* whose functional relevance, however, is yet to be elucidated. In any case, 3′-UTRs sequences are known to be crucial for stability, localization and expression of mRNA, as well as for protein–protein interactions. Therefore, alterations in this region, such as that described herein, hold the potential to affect gene regulation and determine specific pathological conditions^20^. Indeed, several COX1 inhibitors (mimicking the deleterious effect of functional polymorphisms) are anticipated to be potential therapeutic agents for atherosclerosis^21,22^. In any case, the conclusions drawn from this particular set of results are less solid than those regarding the occurrence of CV events, as the reported associations lost statistical significance after Bonferroini correction of the results.

Finally, we also genotyped a group of individuals with normal renal function. Our findings showed that three SNPs in the gene coding for COX2 were associated with a modified risk of nephrosclerosis. In this regard, COX2 levels have been found to be elevated in patients with CKD and have been proposed to contribute to the disease process by mediating vascular calcification in vascular smooth muscle cells, among other mechanisms^23^. One of these three SNPs is rs20417, a G-765C base change in the gene promoter region that has been shown to potentiate COX2 transcription^24^. This effect would translate into a greater synthesis of PGE2 and hence increased inflammatory activity in the kidney compatible with the observed elevated CKD risk. This variant has been widely studied, but, in the renal setting, only one study has investigated its connection with CKD (diabetic nephropathy), albeit with negative results^25^. Of the other two variants, only rs2066826 has reports on its putative clinical impact, having been associated with type 2 diabetes mellitus^26^, atherosclerosis^27^ and cancer^28^. This SNP was also studied in relation to allograft survival in kidney transplant, but no associations were observed^29^. In any case, it should be noted that the improvement caused by these SNPs when added to a ROC model containing standard CKD risk factors was negligible, and hence caution should be exerted when extrapolating these particular results.

Some limitations of the study were that the associations with blood pressure traits or atherosclerosis measurements did not survive Bonferroni correction for multiple testing, or that the definition of CVE was heterogeneous, including coronary heart disease manifestations along with other outcomes. In addition, the low incidence of CVE in the control group prevented genetic associations to be analyzed in these individuals. This could also be behind the small, though significant, improvement of predictive power shown by the combined clinical-genetic model in the whole population of study. Finally, it was difficult in general to link the reported genotype–phenotype associations to a particular consequence of a certain SNP, e.g. altered genetic expression or enzymatic activity. The reason is that we used tag-SNPs in the present study, i.e., intronic variants that account for the genetic variability in an area of the gene locus but that do not normally have a known functional impact. On the other hand, this design allows to point out certain regions of a gene whose variability is key for an outcome without having to screen for all the remaining variants. An additional strength of the study is that the patient cohort was uncommonly homogenous, as nephrosclerosis patients are usually combined with subjects with diabetic nephropathy and other conditions in more heterogeneous CKD groups^30^.

This is the first time to our knowledge that the CV impact of index genetic variants in the cyclooxygenase route is evaluated in nephrosclerosis patients, a condition we think constitutes an ideal setting to study genetic associations with CV traits and events. Overall, our findings highlight the importance of this route in the extremely high CV risk experienced by nephrosclerosis patients and add to the growing body of evidence pointing out the PGE2 synthesis/activity axis as a promising therapeutic target in this field.

## Subjects and methods

### Study subjects

Controls (n = 716) and patients (n = 493) were recruited from three different sources, namely the NEFRONA repository, which is a collection of biological samples available for researchers that was created in a previous project on CV features of Spanish individuals^31^; the Nephrology Service of the Badajoz University Hospital (Badajoz, Spain); and the biobank at the Instituto de Salud Carlos III (ISCIII), which stores DNA samples from Spanish healthy subjects.

Control subjects had to present an eGFR over 60 ml/min/1.73 m^2^ to be enrolled. Inclusion criteria for CKD patients were to be over 18 years of age, to present a stage 3 or higher renal impairment (eGFR < 60 ml/min/1.73 m^2^) and to have histological findings compatible with vascular nephropathy or to meet clinical criteria (advanced age, long-term hypertension, left ventricular hypertrophy, initial mild renal failure and proteinuria < 0.5–1 g/24 h in the absence of other renal disease. On the other hand, the occurrence of a CV event (see definition below) before the start of the study, carotid artery surgery, transplantation, pregnancy, active infection and a life expectancy of less than 12 months were all considered exclusion criteria.

All participants provided written consent for their inclusion. The study was approved by the Ethics Committees of Badajoz University Hospital and the University of Extremadura and was carried out in accordance with the Declaration of Helsinki and its subsequent revisions.

### Clinical variables

Diagnostic and prognostic stratification of patients was conducted with the KDIGO classification and table of progression risk and the CONSORTIUM-CKD equation. Kidney function was assessed by the Modification of Diet in Renal Disease (MDRD) equation. Proteinuria was considered when more than 500 mg protein (or 300 mg albumin) were found in 24-h urine samples. A biopsy was conducted to confirm diagnosis when proteinuria was over 1 g.

A total of 412 out of the 493 patients with nephrosclerosis were examined to identify signs of clinical or subclinical atherosclerosis as previously described^32^. Briefly, explorations were conducted in accordance to the American Society of Echocardiography^33^ and the Mannheim IMT Consensus^34^ with a B-mode ultrasound (Vivid BT09, GE Healthcare, Waukesha, WI, USA). IMT, a marker of subclinical atherosclerosis, was measured in the right and left common carotid arteries and defined as the distance between the leading edge of the lumen intima echo and the leading edge of the media-adventitia echo in the far wall. IMT > 1.5 mm protruding into the lumen was considered atheromatous plaque. ccIMT 24-month progression was also calculated in the nephrosclerosis patients and expressed as mm changed/year. The threshold for accelerated progression was set at the 75th percentile value. Finally, a severity score for atherosclerosis was established based on ccIMT measurements and ankle-brachial index (ABI). A score of 0 was assigned if ccIMT < 90% reference interval and ABI > 0.9; 1 was assigned when ccIMT ≥ 90% reference interval and/or ABI = 0.7–0.9; 2 when there was a carotid plaque with stenosis < 125 cm/seg; and 3 if the stenosis was ≥ 125 cm/seg and/or ABI < 0.7.

Follow-up was set at four years and patients were followed until the earliest of CV event, death, or end of study. CV risk was defined as the likelihood of experiencing a CV event, which included acute myocardial infarction, acute coronary syndrome, coronary catheterization requiring angioplasty, coronary bypass, typical angina with positive stress tests, sudden death, cerebrovascular accident, peripheral arterial disease, aortic aneurysm and lower limb ischemia. CV events were diagnosed by the responsible clinicians at the collaborating hospitals during follow-up; these data were included in the patients’ electronic health records, from where we retrieved them to carry out the present study.

### Genetic analyses

DNA was purified from 10-ml whole blood samples in the case of patients recruited at Badajoz University Hospital, following a standard procedure of phenol–chloroform extraction and ethanol precipitation. Genetic material from biological samples stored at the NEFRONA repository and at the ISCIII biobank was extracted by QIAamp DNA Blood Kits and DNA was stored at 4° until analyzed. Genotyping was carried out at Centro Nacional de Genotipado (CeGen), Madrid, Spain, using a OpenArray customized panel on a QuantStudio™ 12 K Flex Real-Time PCR System (Life Technologies, Carlsbad, California, USA). Quality control was conducted by including sample trios with known genotypes from the Coriell Institute in all the analyses.

The study design called for the identification of tag-SNPs in the five genes of interest, namely *PTGS1* (Accession No. ENSG00000095303), *PTGS2* (ENSG00000073756), *PTGES* (ENSG00000148344), *PTGES2* (ENSG00000148334) and *PTGES3* (ENSG00000110958). For this, we retrieved genetic variability data for European populations from the 1000 genomes project (http://www.internationalgenome.org/) in *vcf* format and created *ped* files with the *vcf to ped converter* tool of Ensembl (https://www.ensembl.org/Homo_sapiens/Tools/VcftoPed). These *.ped* files were then analyzed with Haploview 4.2 software to assign tag-SNPs, considering a pair-wise tagging with r^2^ > 0.8 and a MAF > 0.05. The complete list of 38 SNPs studied, with their corresponding alleles, MAF, and p-values for Hardy–Weinberg equilibrium test are shown in Supplementary Table S4.

### Statistical analyses

Mean and standard deviation (SD) was used to describe parametric variables, whilst median and IQR in parenthesis was used for data not normally distributed. Chi-square tests were utilized to compare categorical variables. Quantitative variables were compared either by t-test or Mann–Whitney (2 groups) or by ANOVA or Kruskal–Wallis (> 2 groups) depending on the data distribution. Genetic associations with clinical variables were assessed by logistic regression adjusting for relevant covariates, namely sex, age, body mass index, ethnicity, diabetes, hypertension or CKD stage^11,35^, which were chosen based on univariate analyses and/or clinical criteria (variables that had previously been associated with CKD). Genetic analyses were carried out under a dominant model of inheritance, i.e., carriers vs. non-carriers, because the resulting genotype groups were the most balanced in terms of size, as we have described in previous CKD studies^11,12^.

CV event-free survival was calculated by Kaplan–Meier curves and the effect of the different genotypes was compared with the log-rank test. Additional Cox regression procedures were carried out to control for classic CV risk factors. The predictive value of the SNPs regarding the risk of nephrosclerosis and CVE was evaluated with ROC curves, which were generated for models with classic risk factors with or without genetic information. The DeLong test was used to detect differences between the area under the curve (AUC) of these models.

Statistical power calculations were conducted considering an arbitrary effect size of 2.0 and a type-1 error of 0.05. With the available sample size, the power to detect genetic associations with the disease ranged from 0.872 to 0.996 for the lowest and highest MAF values, respectively (Quanto software v. 1.2.4, USC, Los Angeles, USA). The threshold for statistically significant associations was set at p < 0.05. Bonferroni correction for the 37 SNPs assayed (one of them was not in Hardy–Weinberg equilibrium) lowered the significance threshold to 0.0013.

The *SNPassoc*, *pROC* and *survival* packages (R software) and IBM SPSS v.22.0 (SPSS Inc., Chicago, IL, v.22.0) were utilized for the statistical analyses.

## Supplementary Information

Supplementary Figure S1.

Supplementary Figure S2.

Supplementary Tables.

## Data Availability

The datasets generated during and/or analyzed during the current study are available from the corresponding author on reasonable request.
