# Supplementary figures and images for: Influence of variability in the cyclooxygenase pathway on cardiovascular outcomes of nephrosclerosis patients
Source: Sci Rep. 2023 Jan 23;13:1253. doi: 10.1038/s41598-022-27343-z (PMC9870986; doi:10.1038/s41598-022-27343-z)

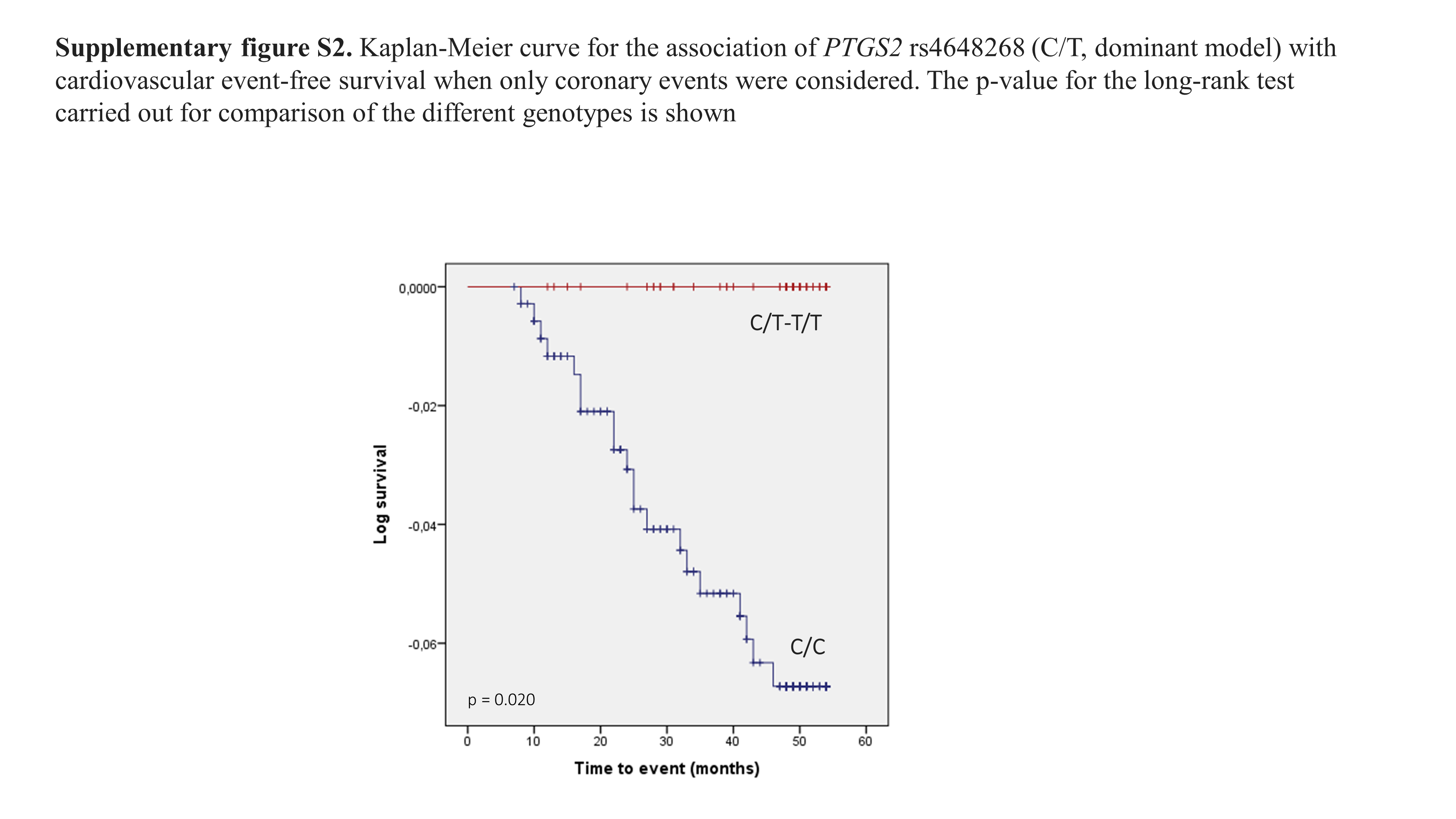

Supplement: Supplementary file 1 — Supplementary Figure S1. [file 41598_2022_27343_MOESM1_ESM.tif]

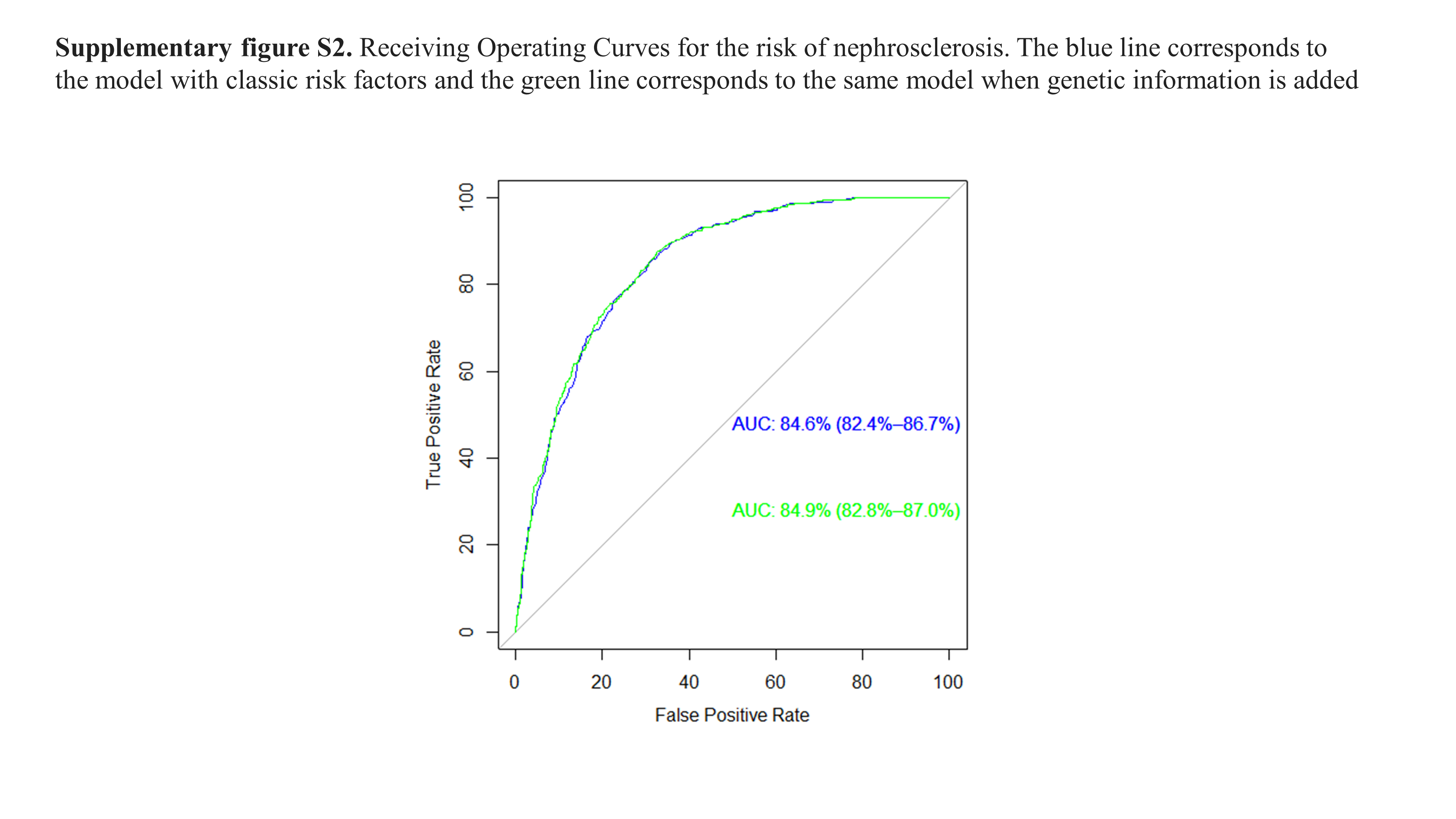

Supplement: Supplementary file 2 — Supplementary Figure S2. [file 41598_2022_27343_MOESM2_ESM.tif]
