# Supplementary material for: Influence of variability in the cyclooxygenase pathway on cardiovascular outcomes of nephrosclerosis patients
Source: Sci Rep. 2023 Jan 23;13:1253. doi: 10.1038/s41598-022-27343-z (PMC9870986; doi:10.1038/s41598-022-27343-z)
Supplement: Supplementary file 3 — Supplementary Tables. [file 41598_2022_27343_MOESM3_ESM.docx]

**Supplementary Tables**

**Supplementary Table S1.** Cox regression analyses model showing the effect of rs4648268 on cardiovascular event-free survival in patients with nephrosclerosis

|  | **B** | **SE** | **Wald** | **HR** | **CI** | **p-Value** |
| --- | --- | --- | --- | --- | --- | --- |
| Sex | -1.14 | 0.43 | 6.99 | 0.32 | 0.14-0.75 | 0.008 |
| Age | 0.06 | 0.02 | 6.99 | 1.07 | 1.02-1.12 | 0.008 |
| BMI | 0.05 | 0.03 | 2.60 | 1.06 | 0.99-1.13 | 0.107 |
| DM | 0.51 | 0.34 | 2.31 | 1.67 | 0.86-3.24 | 0.129 |
| Stage of CKD | 0.45 | 0.23 | 3.66 | 1.57 | 0.99-2.48 | 0.056 |
| Hypertension | 0.46 | 1.02 | 0.20 | 1.58 | 0.21-11.71 | 0.652 |
| rs4648268 | -1.18 | 0.60 | 3.85 | 0.31 | 0.10-1.00 | 0.049 |

BMI, body mass index; DM, diabetes mellitus.

**Supplementary Table S2.** Cox regression analyses model showing the effect of rs2958155 on cardiovascular event-free survival in patients with nephrosclerosis

|  | **B** | **SE** | **Wald** | **HR** | **CI** | **p-Value** |
| --- | --- | --- | --- | --- | --- | --- |
| Sex | -1.13 | 0.42 | 7.27 | 0.32 | 0.14-0.74 | 0.007 |
| Age | 0.05 | 0.02 | 5.35 | 1.05 | 1.01-1.10 | 0.021 |
| BMI | 0.04 | 0.03 | 1.09 | 1.04 | 0.97-1.11 | 0.297 |
| DM | 0.66 | 0.32 | 4.22 | 1.94 | 1.03-3.63 | 0.040 |
| Stage of CKD | 0.38 | 0.23 | 2.88 | 1.47 | 0.94-2.29 | 0.090 |
| Hypertension | -0.03 | 0.74 | 0.001 | 0.97 | 0.23-4.12 | 0.971 |
| rs2958155 | 0.88 | 0.38 | 5.45 | 2.41 | 1.15-5.04 | 0.020 |

BMI, body mass index; DM, diabetes mellitus.

**Supplementary Table S3.** Cox regression analyses model showing the effect of rs11300958 on cardiovascular event-free survival in patients with nephrosclerosis

|  | **B** | **SE** | **Wald** | **HR** | **CI** | **p-Value** |
| --- | --- | --- | --- | --- | --- | --- |
| Sex | -1.07 | 0.42 | 6.59 | 0.34 | 0.15-0.78 | 0.010 |
| Age | 0.05 | 0.02 | 5.67 | 1.05 | 1.01-1.10 | 0.017 |
| BMI | 0.03 | 0.03 | 0.92 | 1.03 | 0.97-1.10 | 0.337 |
| DM | 0.70 | 0.32 | 4.76 | 2.01 | 1.07-3.76 | 0.029 |
| Stage of CKD | 0.35 | 0.23 | 2.41 | 1.42 | 0.91-2.22 | 0.120 |
| Hypertension | -0.10 | 0.74 | 0.02 | 0.90 | 0.21-3.82 | 0.891 |
| rs11300958 | 0.79 | 0.33 | 5.80 | 2.20 | 1.16-4.18 | 0.016 |

BMI, body mass index; DM, diabetes mellitus.

**Supplementary Table S4.** Genetic variants included in the present study

| **rs number** | **Gene** | **Position** | **Ref allele** | **Alternate allele** | **MAF** | **HWE** |
| --- | --- | --- | --- | --- | --- | --- |
| rs7045826 | *PTGS1* | 9:122372881 | G | A | 5.8 | 0.791 |
| rs3119773 | *PTGS1* | 9:122372975 | G | A | 13.2 | 0.900 |
| rs10306122 | *PTGS1* | 9:122373210 | T | C | 7.4 | 0.131 |
| rs1213265 | *PTGS1* | 9:122374168 | T | C | 9.1 | 1.0 |
| rs10306135 | *PTGS1* | 9:122375416 | A | T | 16.5 | 0.529 |
| rs5788 | *PTGS1* | 9:122381513 | C | A | 14 | 0.468 |
| rs1238420 | *PTGS1* | 9:122381866 | G | A | 5.8 | 0.050 |
| rs6478565 | *PTGS1* | 9:122386353 | A | G | 18.6 | 0.697 |
| rs76942325 | *PTGS1* | 9:122389096 | T | G | 6.5 | 0.342 |
| rs10306194 | *PTGS1* | 9:122394919 | C | A | 17.9 | 0.491 |
| rs689470 | *PTGS2* | 1:186671926 | G | A | 3.9 | 0.092 |
| rs2206593 | *PTGS2* | 1:186673297 | G | A | 6.3 | 1.0 |
| rs5275 | *PTGS2* | 1:186673926 | A | G | 28.9 | 0.723 |
| rs2066826 | *PTGS2* | 1:186676795 | C | T | 15.1 | 0.369 |
| rs20432 | *PTGS2* | 1:186677191 | A | C | 18.9 | 0.398 |
| rs4648268 | *PTGS2* | 1:186678006 | C | T | 9.6 | 0.499 |
| rs5277 | *PTGS2* | 1:186679065 | C | G | 19.2 | 0.159 |
| rs20417 | *PTGS2* | 1:186681189 | C | G | 19.7 | 0.524 |
| rs689466 | *PTGS2* | 1:186681619 | T | C | 19.1 | 0.257 |
| rs2745559 | *PTGS2* | 1:186682870 | C | A | 19.3 | 0.926 |
| rs75051660 | *PTGS2* | 1:186804773 | G | T | 3.3 | 0.636 |
| rs45544737 | *PTGES* | 9:129738701 | G | A | 5.2 | 0.553 |
| rs2302821 | *PTGES* | 9:129739602 | A | C | 8.5 | 0.134 |
| rs4837404 | *PTGES* | 9:129743380 | A | G | 34.2 | 0.948 |
| rs11790782 | *PTGES* | 9:129744368 | G | A | 8.8 | 0.361 |
| rs2241270 | *PTGES* | 9:129752610 | C | T | 13.9 | 0.810 |
| rs2241271 | *PTGES* | 9:129752802 | T | C | 23.4 | 0.423 |
| rs11792431 | *PTGES* | 9:129752968 | G | A | 6.9 | 0.818 |
| rs13283456 | *PTGES2* | 9:128122474 | C | T | 20.5 | 0.928 |
| rs884115 | *PTGES2* | 9:128125577 | C | T | 14 | 0.399 |
| rs2958155 | *PTGES3* | 12:56670761 | G | T | 38.8 | 0.855 |
| rs61939899 | *PTGES3* | 12:56676467 | T | C | 8.4 | 0.848 |
| rs11300958 | *PTGES3* | 12:56678106-7 | - | G | 28.6 | 0.943 |
| rs73113847 | *PTGES3* | 12:56682211 | C | T | 2.8 | 0.056 |
| rs11171933 | *PTGES3* | 12:56682782 | G | A | 4.7 | 0.106 |
| rs12824563 | *PTGES3* | 12:56684044 | A | G | 5.2 | 0.240 |
| rs78343990 | *PTGES3* | 12:56685251 | T | C | 6.9 | 0.039 |
| rs17445108 | *PTGES3* | 12:56688274 | G | A | 9.4 | 0.866 |

Ref, reference; MAF, minor allele frequency; HWE, p-value for Hardy-Weinberg Equilibrium test
